# Bridging the gap: technology-driven solutions to overcome food insecurity and improve nutrition access for older adults

**DOI:** 10.3389/fpubh.2026.1847218

**Published:** 2026-07-07

**Authors:** Claudia Reytor-González, Martín Campuzano-Donoso, Maritza Aguirre Munizaga, Oki Yonatan Oentiono, Janeth Castano Jimenez, Martha Montalvan, Daniel Simancas-Racines

**Affiliations:** 1 Facultad de Ciencias de la Salud y Bienestar Humano, Universidad Tecnológica Indoamérica, Ambato, Ecuador; 2 Facultad de Ciencias Agrarias, Universidad Agraria del Ecuador, Guayaquil, Ecuador; 3 Mayapada Kuningan Hospital, Jakarta, Indonesia; 4 Department of Emergency Medicine, Indiana University School of Medicine, Indianapolis, IN, United States; 5 Escuela de Medicina, Universidad Espíritu Santo, Samborondón, Ecuador

**Keywords:** assistive technology, digital health, food insecurity, healthcare, older adults, social isolation

## Abstract

Food insecurity and social isolation are interrelated challenges affecting many older adults, with overlapping pathways that include limited income, reduced mobility, difficulty shopping or preparing meals, loss of shared mealtime routines, and lack of practical support for food procurement. These pathways may contribute to poorer dietary intake, malnutrition risk, deteriorating health, and reduced quality of life. As the global population ages, digital health and assistive technologies may support selected aspects of nutrition access, care coordination, and social connectedness, although their effects depend on implementation context and the availability of human and community support. This review explores the potential of tools such as wearable devices, telehealth services, virtual community centers, GIS mapping, and mobile markets in addressing these challenges. It also highlights the importance of integrating caregivers and coordinated care teams into technology-driven interventions to improve outcomes for older adults with limited access to traditional services. While these innovations present exciting opportunities, barriers such as the digital divide, limited digital literacy, data privacy concerns, and the risk of widening existing health disparities must be addressed. Ensuring that technologies are designed inclusively, ethically, and with input from older users is essential for building scalable, sustainable solutions that truly enhance the quality of lives of aging individuals. As digital health continues to evolve, it must prioritize equity, accessibility, and human-centered design to effectively support aging in place and reduce the burden of food insecurity and social isolation.

## 1 Introduction

Food insecurity (FI), defined as the limited or uncertain availability of nutritionally adequate and safe foods, is a growing public health concern, particularly among older adults (1, 2). According to the Food and Agriculture Organization of the United Nations, food security exists when all people, at all times, have physical, social, and economic access to sufficient, safe, and nutritious food that meets their dietary needs and preferences for an active and healthy life (3). Among older adults, FI is more than a matter of hunger; it directly compromises nutritional status and overall health, contributing to malnutrition, functional decline, and reduced quality of life (4–7). Despite being historically underrepresented in FI research and policy efforts due to lower perceived prevalence, the number of food-insecure older adults has doubled since the early 2000s (8, 9).

This rise is particularly troubling because food-insecure older adults are significantly more likely to experience multiple chronic conditions, including diabetes, hypertension, heart disease, and depression (9, 10). The challenges they face are often multifaceted. Financial hardship, which is worsened by limited fixed incomes, rising medical expenses, and inadequate safety net programs, is a central barrier (11, 12). Additionally, physical limitations, such as mobility impairments or poor vision, can make it difficult to shop for and prepare food, while cognitive decline and social isolation further compound access issues (13–15). Social isolation may worsen FI through several mechanisms, including reduced assistance with grocery shopping, transportation, meal preparation, and benefit navigation, as well as the loss of shared meals that often structure appetite, food choice, and dietary variety in later life (16–18). Conversely, FI may reinforce isolation when financial constraints, shame, poor health, or mobility limitations reduce participation in social and community activities (16, 18). In 2020 alone, nearly 7% of households with older adults, and over 8% of older adults living alone, were food insecure in the United States (14), and the situation is often more severe in low-income, rural, or marginalized communities.

Addressing FI in aging populations requires innovative solutions that go beyond traditional service models. Long-standing non-digital and community-based interventions, such as home-delivered meals, congregate meal programs, and mobile food services, remain central to supporting food access among older adults. In this review, these models are used as important points of comparison and, when relevant, as examples of hybrid interventions that may incorporate digital tools for referral, coordination, benefit navigation, delivery logistics, or social engagement. As digital tools become increasingly integrated into daily life, technology presents a promising avenue to bridge longstanding gaps in food access and nutrition monitoring (19). While much of the early research and development in health-related technologies has focused on younger populations, there is growing recognition of the need to adapt and expand these tools for older adults, a field known as gerontechnology (19). Technology offers several advantages: it can enable remote dietary assessments, support chronic disease management, deliver tailored nutrition education, and facilitate food delivery and caregiving coordination, all while accommodating the preferences and limitations of older users (20).

This narrative review aims to critically examine technology-driven strategies that may support food security, nutrition access, and social connectedness among older adults. Rather than evaluating technology as a standalone solution, the review considers digital and technology-enabled interventions as components of broader care, community, and policy systems. Specifically, the review addresses four guiding questions: (1) what individual, social, and structural factors link FI, nutrition access, and social isolation in later life?; (2) what digital or technology-enabled strategies have been described to support nutrition access among older adults?; (3) what evidence supports these strategies, and where is the evidence direct, indirect, or still emerging?; and (4) what ethical, geographic, infrastructural, caregiver-related, and policy constraints shape the feasibility and equity of implementation? By addressing these questions, this review seeks to provide a balanced synthesis of both the potential and the limitations of technology-supported approaches for older adults experiencing or at risk of FI.

## 2 Methods

This article was designed as a narrative review rather than a systematic review or meta-analysis. The purpose was to synthesize and critically interpret literature on FI, nutrition access, social isolation, and technology-supported interventions among older adults, with particular attention to implementation, equity, and ethical considerations. The reporting of this narrative review was informed by general principles for transparent narrative reviews, including clear justification of scope, search approach, referencing, and synthesis (21).

A targeted literature search was conducted using PubMed/MEDLINE, Google Scholar, and relevant institutional or policy sources. Search terms included combinations of: “FI,” “food security,” “nutrition access,” “malnutrition,” “older adults,” “aging,” “social isolation,” “loneliness,” “digital health,” “telehealth,” “telemedicine,” “mHealth,” “mobile applications,” “wearables,” “remote monitoring,” “assistive technology,” “smart kitchen,” “geographic information systems,” “GIS,” “food delivery,” “mobile markets,” “caregivers,” “digital divide,” and “health equity.” Reference lists of relevant articles were also screened to identify additional studies and reports.

Eligible sources included peer-reviewed original studies, systematic reviews, scoping reviews, narrative reviews, qualitative studies, implementation studies, and selected policy or grey-literature documents. Grey literature was included when it provided information not readily available in peer-reviewed articles, such as program eligibility criteria, service delivery models, federal or community nutrition infrastructure, benefit-navigation tools, public-private partnerships, or digital implementation strategies. These sources were used to describe policy context, program design, implementation mechanisms, and service availability, but not as primary evidence of clinical effectiveness unless they summarized formal evidence reviews or evaluations.

Technology-driven strategies were defined broadly as digital, assistive, data-enabled, or technology-supported approaches intended to improve food access, nutrition monitoring, dietary support, social connection, care coordination, or service delivery. This included telehealth and telenutrition, mobile health applications, wearable devices, remote monitoring systems, smart kitchen technologies, GIS-based food mapping, online or app-supported food delivery, virtual community platforms, and digitally supported public or community programs. Traditional non-digital interventions, such as home-delivered meals or mobile markets, were included only when they served as comparators, contextual examples, or hybrid models incorporating digital components.

No formal geographical restriction was applied. However, because digital infrastructure, health-system capacity, food assistance programs, social care models, and cultural attitudes toward technology vary considerably across countries and regions, geographical transferability was considered during interpretation. The literature identified and discussed in this review was unevenly distributed, with many empirical studies, implementation examples, and policy initiatives originating from high-income and Western settings, particularly the United States, the United Kingdom, and other digitally resourced health systems. Evidence from non-Western and low- and middle-income settings was included when available, particularly for food insecurity prevalence, mental health, mobile health, and food system challenges. Nevertheless, the predominance of high-income settings limits the extent to which findings can be generalized to regions with different connectivity, workforce capacity, reimbursement structures, food assistance systems, or community-care infrastructure.

The synthesis was organized thematically rather than statistically. Findings were grouped into domains covering determinants of FI in later life, technology-based strategies, community-based technology initiatives, caregiver and health-system integration, ethical challenges, and future implementation priorities. Throughout the review, the strength and applicability of evidence were considered by distinguishing direct evidence in older adults or food-insecure populations from indirect evidence extrapolated from other populations or settings, and from expert, policy, or implementation-based recommendations.

Because this was a narrative review, we did not conduct a formal bibliometric analysis by country of origin. However, during revision we reviewed the geographical distribution of the most frequently discussed studies and examples. This confirmed that the evidence base is concentrated in high-income settings, while non-Western and low- and middle-income evidence remains more limited and is often focused on prevalence, mental health, or broad food system determinants rather than technology-supported nutrition interventions for older adults. We therefore avoid presenting the findings as globally generalizable and instead discuss transferability as context-dependent.

## 3 Understanding food insecurity among older adults

FI among older adults is a growing concern that reflects broader demographic, socioeconomic, and health-related trends. In the United States, rates of FI among this population more than doubled between 2007 and 2016, rising from 5.5 to 12.4%, with disproportionately high rates among low-income individuals (22). Globally, the issue is set to intensify due to rapid population aging. By 2030, one in six people worldwide will be over age 60, and by 2050, the number of people aged 80 or older is expected to triple, reaching 426 million (23). Without targeted interventions, the number of older adults living in food-insecure households is expected to continue rising (9). Recent estimates show that 6.9% of U. S. households with at least one adult aged 65 or older, and 8.3% of single-person households with an older adult, are food insecure (14). Older adults make up over 20% of all food-insecure households in the country (14). This is especially concerning given that over 80% of older Americans have at least one chronic health condition (24, 25), and more than a third experience difficulties with everyday tasks like preparing meals, further complicating food access and dietary consistency (25), and overall quality of life (1, 11, 15).

These figures should be interpreted not only as indicators of inadequate food access, but also as markers of cumulative disadvantage in later life (9). FI among older adults often emerges at the intersection of constrained income, high healthcare and medication costs, disability, transportation barriers, social isolation, and limited access to age-appropriate food assistance. These factors interact dynamically: poor health can reduce the ability to obtain and prepare food, while inadequate nutrition can worsen chronic disease, functional decline, and social participation (1, 11, 15). A critical understanding of FI in later life therefore requires moving beyond isolated risk factors toward a systems perspective that considers economic, clinical, geographic, and relational determinants together.

### 3.1 Socioeconomic contributors

FI in older adults is strongly linked to economic hardship, but income alone does not fully explain vulnerability. Limited household income often interacts with rising food prices, housing costs, transportation needs, healthcare expenses, and out-of-pocket medication costs, creating trade-offs between food, medical care, utilities, and other basic needs (15, 26). Evidence from older adult populations shows that food insecurity is associated not only with low income but also with physical and economic barriers to food access, lower educational attainment, living alone, and racial or ethnic inequities that reflect broader structural disadvantage (9, 11).

Multiple socioeconomic factors such as poverty, limited education, racial/ethnic disparities, and living alone are significantly associated with higher FI rates among older individuals (11). For example, the findings from the NHANES study underscore that individuals with a poverty-income ratio below 1.85, those never married, or individuals without a college education are more likely to experience food access limitations. Non-Hispanic Black and Hispanic populations are also disproportionately affected, reflecting longstanding structural inequalities in food systems and social support (11). In many cases, the high cost of prescription medications and healthcare leaves little income available for food purchases (27), while chronic disease-related physical impairments further limit food procurement and preparation (1). Conversely, food assistance may be available but underused because of stigma, administrative complexity, immigration concerns, language barriers, or lack of digital access. This distinction is important for technology-based interventions: apps, delivery platforms, or benefit-navigation tools may reduce selected access barriers, but they cannot compensate for insufficient income, inadequate benefits, or unaffordable food environments unless linked to broader social protection and food assistance policies.

### 3.2 Health-related contributors

The relationship between FI and health in older adults is reciprocal and compounding (28). On one hand, chronic illness and disability can reduce the ability to acquire and prepare food, leading to poor dietary intake. On the other, inadequate nutrition exacerbates existing health conditions (1, 15, 29). Mental health is also affected: food-insecure older adults are more than twice as likely to experience depression, which in turn can reduce appetite, energy levels, and motivation to maintain healthy eating habits (10, 30). A systematic review focusing on low- and middle-income countries found consistent associations between FI and deteriorating mental health, with variations based on sex, age, residence, and physical health status (31).

This reciprocal relationship is particularly important because much of the evidence is observational. Associations between food insecurity and diabetes, cardiovascular disease, depression, disability, or poor self-rated health should not be interpreted as simple one-directional causality (30, 32). Rather, food insecurity may operate as both a cause and a consequence of poor health, mediated by income constraints, healthcare expenditures, medication trade-offs, dietary quality, psychological stress, and reduced functional capacity. This framing has practical implications: interventions that only provide nutrition education may be insufficient when poor diet quality is driven by poverty, disability, lack of transportation, or inability to prepare meals.

### 3.3 Geographic disparities

Geography shapes food insecurity through the distribution of food outlets, transportation systems, healthcare services, broadband access, and community-based supports. Rural older adults may face particular barriers because grocery stores, dietitians, public transportation, food delivery infrastructure, and social services are often more dispersed, while disability or inability to drive can further restrict access (33, 34). However, geographic disadvantage is not limited to rural areas. Urban older adults may also experience FI when affordable healthy foods are unavailable, physically inaccessible, culturally inappropriate, or economically out of reach (35).

These geographic patterns matter for technology-based interventions because digital solutions depend on place-based infrastructure. Online grocery ordering, telehealth, remote monitoring, and benefit navigation tools require broadband connectivity, delivery networks, accessible devices, and local service capacity. In regions where these infrastructures are weak, technology may have limited reach unless combined with transportation support, community hubs, telephone-based services, mobile markets, or home-delivered meals (36, 37). Thus, geography should be understood not only as distance to food stores but also as the spatial organization of care, connectivity, and social support.

### 3.4 Consequences of food insecurity

The effects of FI on older adults are wide-ranging and severe. Food insecure individuals are at increased risk of chronic conditions including diabetes, cardiovascular disease, respiratory illnesses, obesity, and depression (1, 15, 38). They also report more physically unhealthy days, greater disability, lower quality of life, and higher rates of co-morbidity (38–45). Longitudinal research indicates a bidirectional relationship between FI and healthcare utilization. One study found that older adults experiencing FI were 40% more likely to be hospitalized at the same time point, with depression accounting for nearly one-fifth of that association. Furthermore, a prior hospitalization increased the likelihood of becoming food insecure two years later by 50% (40).

### 3.5 Social isolation, meal practices, and cognitive vulnerability

Social isolation is not only a parallel geriatric concern but also a pathway through which FI and poor nutrition may develop or worsen. Older adults who live alone or lack regular social interaction may be less likely to cook complete meals, maintain dietary variety, or participate in shared mealtime routines that support appetite and food intake (17). Social networks also provide practical support for transportation, grocery shopping, meal planning, food preparation, and navigation of food assistance programs; when these networks are absent, both perceived and actual FI may increase (16).

The relationship is likely bidirectional. FI can contribute to poorer physical and mental health, while depression, loneliness, mobility limitations, and cognitive decline may reduce motivation or capacity to obtain and prepare food (16, 18). In this context, social isolation may act as both a determinant and a consequence of FI: limited resources and poor health can restrict social participation, while reduced social participation can further limit access to food-related support. This interaction is especially relevant for older adults with chronic disease or functional limitations, who may experience rapid changes in their ability to shop, cook, eat independently, or seek assistance (24).

Therefore, interventions for FI in later life should not be evaluated only by their ability to deliver food or nutrition information. Their potential contribution to social connection, caregiver involvement, dignity, autonomy, and linkage to community resources is also important. However, evidence directly testing whether technology-supported interventions simultaneously improve food security and reduce social isolation remains limited, and findings should be interpreted with caution.

## 4 Current technology-based strategies addressing nutrition access

Before reviewing specific strategies, it is important to emphasize that technology should not be understood as a stand-alone solution to FI or social isolation in later life. FI is shaped by income, housing, transportation, disability, social support, food prices, local food systems, and public policy; therefore, digital tools can only be effective when embedded within broader social, clinical, and community infrastructures. Critical work on telecare and care technologies has cautioned against “silver bullet” framings, in which digital innovation is presented as a simple response to complex care problems (46). In this review, technology-driven strategies are therefore interpreted as potential enablers of access, monitoring, coordination, and connection, rather than substitutes for adequate food assistance, social care, human support, or structural policy interventions (46, 47).

For this reason, the strategies discussed below are considered according to the specific barriers they may address. Some tools primarily support nutrition access or dietary monitoring, such as telenutrition, mobile applications, food delivery platforms, and wearables. Others primarily address geographic or service-access barriers, such as GIS mapping, mobile markets, and digitally assisted benefit navigation. A smaller subset may also support social connection, including virtual community platforms, caregiver-linked monitoring, congregate meal programs with digital coordination, and community-based interventions that preserve shared food practices (48–50). Distinguishing these functions is important because an intervention that improves food access does not necessarily reduce loneliness, and an intervention that increases social contact does not necessarily improve food security.

### 4.1 Telehealth and remote nutrition counseling

Telehealth has become a valuable resource of modern healthcare, particularly following the COVID-19 pandemic, which catalyzed its rapid expansion across various clinical settings (51, 52). Although telemedicine cannot fully replace in-person clinical visits, it may serve as a valuable complementary modality for certain types of healthcare consultations. In particular, telemedicine can faciliatet follow-up appointments, routine monitoring, health education, and consultations that do not require physical examinations. Furthermore, it may help overcome geographical barriers and reduce healthcare-related costs, thereby improving accessibility and convenience for patients (53, 54). The pandemic drove widespread adoption of virtual consultations, remote monitoring, and digital health platforms, especially as many ambulatory services were closed and elective procedures postponed (55–58).

For older adults, the pandemic exacerbated pre-existing health disparities. In a survey of 1,482 seniors across rural Indiana, researchers found that FI, physical health, and loneliness were closely linked pre-pandemic, with post-pandemic data showing increases in FI and loneliness, and physical health remaining a key predictor of food security (1, 59). Telehealth emerged became an important access pathway during this period, especially for vulnerable, food-insecure populations.

Studies also suggested that telemedicine might have potential in supporting chronic disease management. For instance, a mobile app for older Chinese adults with type 2 diabetes significantly improved glycemic control, highlighting the benefits of user-friendly mobile health (mHealth) systems for older populations (60).

Teleconsultations with dietitians offer significant promise in improving dietary behaviors, particularly in low-income and rural communities where access to nutrition professionals is often limited (32, 61–63). A survey of 300 Israeli clinical dietitians during the pandemic revealed a marked shift toward phone (72%) and online (53.5%) consultations, though many practitioners reported limited experience and technological challenges that hindered care quality (64). Despite this, systematic reviews suggest that telehealth interventions can successfully improve diet quality, especially when targeting whole-food dietary patterns, though effects on physiological measures control, such as blood pressure and HbA1c, remain moderate (65).

However, the effectiveness and acceptability of telehealth among older are constrained by barriers such as low digital literacy, sensory and cognitive impairments, limited device access, unreliable connectivity and general distrust in technology (66, 67). Common challenges include small screen sizes, complex navigation systems, and low confidence using mobile devices. Age-appropriate design and training are necessary but not sufficient. Sustained technical support, caregiver or community assistance when desired, affordable connectivity and non-digital alternatives remain essential to avoid excluding older adults who are unable or unwilling to use telehealth-based services (67).

### 4.2 Mobile applications and meal planning platforms

Mobile health applications integrating features such as dietary intake monitoring (i.e., food logging and energy tracking), nutrient profiling systems, health tips and personalized recommendations (68, 69), are increasingly used to deliver behavioral health interventions, particularly as older adults become more engaged in self-managing their health (70, 71). These apps can passively collect health-related data, including physical activity, sleep, and mobility, providing insights into overall well-being (72, 73).

Evidence supports their efficacy in improving dietary habits. A randomized controlled trial using the *Vegethon* app significantly increased vegetable intake among overweight adults (74). Another bilingual app targeting food pantry users improved both the frequency and variety of vegetable consumption in low-resource households (75). Similarly, app-based inhibitory control (IC) training was shown to enhance cognitive restraint and reduce unhealthy food intake (76).

Long-term behavior change through apps is achievable when users are highly engaged. A 12-month trial found that participants who consistently used a Mediterranean diet and physical activity app maintained healthier behaviors better than those receiving lifestyle counseling alone (77). In a longitudinal study of users of the Foodsmart telehealth and nutrition platform, some food-insecure participants improved their food security status and self-reported nutritional status over time (78). Because the study was observational and based on platform users rather than a randomized sample of food-insecure older adults, these findings should be interpreted as promising but indirect evidence rather than proof of effectiveness in the target population. Finally, app-based nutritional intervention has been also proved effective in nudging towards healthier and more sustainable food choices at worksite canteen (79), being important for adults and older adults not only in terms of chronic diseases prevention but also daily performance.

Apps for grocery ordering, meal delivery, and dietary tracking, such as Instacart and Uber Eats may reduce selected access barriers by facilitating food procurement for older adults with mobility, transportation, or caregiving constraints (80, 81). However, their relationship with social isolation is more complex. While delivery platforms can reduce the need for physically demanding shopping trips, they may also decrease opportunities for in-person interaction if used as substitutes for community meals, shared shopping, or social food practices (50). For this reason, app-based food access strategies should be paired, when appropriate, with telephone support, caregiver involvement, community outreach, or social programming rather than assumed to reduce loneliness directly.

Despite the popularity of food delivery apps, usage among older adults remains limited. While ease of use, perceived usefulness, and physical constraints influence adoption, most studies focus on younger populations (82). Evidence from urban China during COVID-19 suggests that online food delivery platforms contributed to short-term food system resilience during lockdowns and may have influenced food purchasing patterns (83). However, this evidence is context-specific and should not be generalized to older adults in settings without comparable delivery infrastructure, digital access, or platform penetration.

Mobile apps are also increasingly tailored for chronic disease management. Data from NHANES show a 10% prevalence of type 2 diabetes in mildly food-insecure individuals (84). Among rural older adults with diabetes, those experiencing FI had higher BMIs and lower income, yet both insecure and secure groups responded similarly to telemedicine-based nutrition counseling (32). Although most app-based interventions target diabetes, they have shown statistically significant reductions in HbA1c in both short and long terms, underlining the potential for broader chronic disease applications (85).

Some apps are designed specifically for older users, offering features like real-time communication with caregivers and biometric monitoring of blood pressure and weight, coupled with professional feedback (86). Nevertheless, challenges such as limited device access, low digital literacy, and affordability persist, disproportionately affecting older, less educated individuals (87, 88). Integration of these tools is most appropriate when applications are accessible, affordable, culturally acceptable, and linked to human support. Importantly, app-based strategies should complement rather than replace telephone-based services, in-person counseling, home-delivered meals, and community programs, particularly for older adults who prefer low-technology interactions or who face cognitive, sensory, financial, or connectivity barriers.

### 4.3 Smart kitchens and assistive technologies

Smart kitchens and Internet of Things (IoT)-enabled devices are increasingly used to assist older adults with food-related tasks. Innovations such as smart refrigerators, voice-activated assistants (like Alexa), and automated grocery lists help manage food storage and preparation while reducing waste and promoting healthy eating (89, 90).

For older adults with cognitive impairments, digital displays and augmented reality tools can support task completion and reduce caregiver burden, particularly in communal living environments (91). Research by Blasco et al. explored the use of smart kitchens to simulate real-life scenarios like preparing meals and dishwashing, highlighting their potential for supporting independent living (92). Additionally, these systems can offer calorie- and nutrition-aware meal planning, assisting in dietary adherence (93, 94). Video analyses of older adults cooking revealed that assistive kitchen technologies could provide both physical and cognitive support, aiding in the design of intuitive, voice-based user interfaces tailored for aging populations (95).

### 4.4 Wearables and nutrient intake monitoring

Wearable technologies are rapidly evolving as powerful tools for monitoring nutrition-related health metrics, particularly among older adults. These devices, ranging from fitness trackers and smartwatches to more advanced biosensors, collect a wide range of physiological and behavioral data that can inform dietary planning and support chronic disease management (96, 97). Commonly monitored parameters include step count, heart rate, energy expenditure, sleep quality, and GPS-based movement patterns, all of which provide contextual insights into an individual’s health status and nutritional needs (98).

More advanced wearables incorporate biosensors capable of detecting hydration levels, blood glucose, ketones, sodium, and even micronutrient markers through sweat, interstitial fluid, or saliva, though many of these remain in early stages of development or clinical validation (98, 99). For instance, continuous glucose monitoring systems have become particularly valuable for diabetic patients, providing real-time data that enable adaptive dietary adjustments and improved glycemic control (100). Similarly, wearable devices that include photo-assisted dietary logs or use computer vision to identify and quantify food intake are emerging, offering passive and objective tracking of dietary behaviors (101). Table 1 provides a structured overview of the types of wearable devices available and their specific relevance to nutrition monitoring.

**Table 1:** Digital and wearable tools with potential relevance to nutrition monitoring.

Device type	Key data collected	Relevance to nutrition monitoring
Activity trackers (96)	Steps taken, distance traveled, calories burned, physical activity levels.	Estimates energy expenditure, informs calorie and macronutrient recommendations.
Smartwatches (96, 97)	Heart rate, GPS, physical activity, sleep patterns, general health metrics.	Monitors stress/recovery impacting nutrition; provides context for energy balance and lifestyle behaviors.
Biosensor patches (98)	Glucose levels, hydration status, electrolytes, hormones (cortisol).	May monitor selected biomarkers such as glucose, hydration-related measures, or electrolytes; clinical validity and nutrition-specific utility vary by sensor and condition.
Camera-based devices / smart glasses (156–158)	Food images, chewing/swallowing gestures, meal context.	May support recognition of eating episodes or estimation of food type and portion size; accuracy depends on image quality, algorithms, user context, and validation against dietary assessment methods.
Saliva-based sensors (159, 160)	Ions, urea, uric acid, cholesterol, creatine, fatty acids, protein, hormones.	May provide experimental or emerging information on selected metabolic or biochemical markers; routine use for nutrition assessment in older adults requires further validation.
Necklace-like sensors (161, 162)	Acoustic inputs from the throat (eating sounds)	May detect eating episodes, chewing/swallowing patterns, or acoustic signals relevant to dysphagia screening; clinical interpretation requires validation and professional follow-up.
Diet tracking/food logging apps/non-werable digital tools (70, 86, 163–165)	User-inputted dietary information.	Provides insight into eating habits; used for personalized coaching and recommendations.

Artificial intelligence and machine learning are increasingly embedded within these platforms, allowing wearables to generate personalized dietary recommendations based on real-time data such as activity level, metabolic rate, and glycemic response (102, 103). For older adults, these AI-supported systems offer a practical means of adapting nutrition plans to evolving needs related to aging (52, 104, 105).

Despite their promise, the widespread adoption of wearables in older populations is tempered by challenges such as limited digital literacy, affordability, and physical usability. However, clinical trials consistently report high compliance when these devices are introduced with sufficient training and support (19, 106, 107). Additionally, wearable-integrated systems can serve as part of broader coaching programs, alerting caregivers or healthcare providers to nutritional risks like undernutrition or unintended weight loss, thereby playing a role in early intervention and prevention strategies (102, 108). However, the monitoring and interpretation of health-related information should be conducted under the guidance of qualified healthcare professionals to ensure accuracy, safety and appropriate clinical decision-making.

### 4.5 Integration with caregivers and remote monitoring by healthcare teams

The integration of telehealth systems, remote monitoring, caregivers, and healthcare teams may strengthen nutrition support for older adults, particularly in rural or underserved settings where access to dietitians and geriatric services is limited (32). Caregivers, including family members, friends, neighbors, and paid care workers, often provide practical support for grocery shopping, meal preparation, medication management, transportation, and recognition of functional or nutritional decline (88). For this reason, caregiver-linked technologies may improve communication, support earlier recognition of risk, and help coordinate nutrition-related care when embedded within clear clinical or community workflows (109, 110).

However, these technologies should not be assumed to reduce caregiver burden. Technology-based interventions may decrease some forms of burden by improving access to information, professional support, or care coordination, but they may also redistribute work onto caregivers through alert management, device maintenance, troubleshooting, data interpretation, appointment coordination, and communication with healthcare teams (47, 109). Critical analyses of technology in care systems emphasize that digital tools can reshape or intensify care roles rather than simply replace or reduce them (47). Therefore, remote monitoring should include explicit assessment of caregiver capacity, consent, digital literacy, availability, and emotional burden before assigning monitoring responsibilities. (111, 112).

Evidence on caregiver outcomes remains heterogeneous. A recent systematic review and meta-analysis of randomized trials found that technology-based interventions may reduce burden among informal caregivers of older adults, but effects varied by intervention format, care-recipient condition, and study quality (113). Accordingly, caregiver-linked nutrition technologies should be implemented with safeguards such as manageable alert thresholds, escalation protocols, training, technical support, and options for caregivers to decline or adjust their role. In the context of FI, these systems are most appropriate when they connect monitoring data to actionable resources, such as dietitian referral, home-delivered meals, food assistance programs, transportation support, or community outreach, rather than merely shifting responsibility for unmet needs onto families.

## 5 Community-based technology initiatives

Technology-enabled community initiatives have emerged as potentially useful approaches in addressing FI, social isolation, and limited mobility among older adults. These programs integrate Geographic Information Systems (GIS), mobile food access solutions, virtual platforms, and public-private partnerships to improve nutrition equity and engagement for older populations, particularly in underserved regions. GIS are central to identifying food deserts, areas with limited access to affordable, nutritious food, and designing data-driven interventions to address these gaps (114). By analyzing the distribution of retail food outlets in relation to residential locations, GIS tools can assess population density, distance to the nearest food source, and neighborhood demographics, enabling precise visualization of food access barriers (50). This spatial modeling can also account for transportation time and physical mobility limitations, enhancing efforts to plan and evaluate targeted solutions such as mobile food delivery or community-supported agriculture programs (115). Importantly, the technology’s ability to integrate diverse data layers supports the strategic allocation of resources, policy monitoring, and the development of equitable nutrition initiatives (116). GIS-based measurements complement self-reported perceptions of the food environment, providing a holistic understanding of both objective and subjective barriers to food access (116–118). This dual-layered assessment is particularly valuable in informing community-based strategies tailored to the specific needs and limitations of older adults.

Mobile farmers markets represent an increasingly used community-based intervention, particularly for older adults facing transportation challenges or physical limitations. These markets bring fresh, affordable produce directly into neighborhoods, thereby reducing the physical, financial, and psychological burdens associated with traditional food procurement (119, 120). Beyond simply increasing food access, these programs also reduce FI by creating informal gathering spaces where older adults can interact, socialize, and build community (121). In Wake County, North Carolina, mobile markets are combined with food deliveries and senior gardening programs, blending practical services with light-touch technology. These initiatives are supported by the Atlanta Regional Commission’s mini-grants, which fund local innovations such as an Assistive Technology Lab and app-assisted grocery delivery through Instacart and Uber. These adaptable, community-driven solutions demonstrate how targeted funding and simple tech tools can be used to address the specific needs of older adults in localized, meaningful ways (122).

These examples illustrate why community-based interventions may be particularly relevant to the intersection between FI and social isolation. Unlike purely transactional food delivery models, mobile markets, congregate meal sites, senior gardening programs, and shared cooking or food education initiatives may combine food access with social participation (48, 119, 121). Nevertheless, the social benefits of these programs depend on local design, transportation, cultural acceptability, staffing, and sustained funding. Digital tools can support scheduling, outreach, benefit navigation, or delivery logistics, but the relational component usually depends on trusted community actors and opportunities for meaningful interaction (49).

Home-delivered meal programs, including Meals on Wheels (MoW), provide an important example of a long-standing community-based response to FI among older adults. Although these programs are not inherently digital interventions, they are relevant to technology-supported food access because digital tools may be used to support referral pathways, volunteer coordination, route planning, eligibility screening, wellness checks, and communication with caregivers or service providers. Evidence suggests that home-delivered meal services can support nutritional needs while also offering social contact and practical assistance for older adults who face sensory, mobility, or cognitive barriers to food access (48, 123). This is consistent with policy and evidence-review guidance identifying home-delivered meals as a strategy to reduce malnutrition among independently living older adults, while also recognizing that meal delivery can provide wellness checks and social contact for homebound individuals (124). Furthermore, ethnographic research in the UK emphasizes that MoW services help older adults overcome sensory and mobility-related food access challenges while fostering meaningful social connections (48). Their inclusion in this review therefore serves not as an example of an app-based intervention, but as a comparator and hybrid model illustrating how technology may strengthen, rather than replace, established community food systems.

Technology-enhanced community programs, such as Food Classes for Older Adults (FCOA), further contribute to nutritional and social wellbeing. These initiatives provide cooking education and promote commensality (shared eating experiences), while addressing common barriers among older adults such as inability to cook (22.7%), shop (31.4%), or manage food expenses (14.6%) (125). When delivered through apps or virtual platforms, these programs become more accessible and effective, facilitating social connectedness and reinforcing healthy dietary habits (49, 126).

Virtual community platforms may extend some community-based services by offering online group activities, wellness checks, nutrition education, or caregiver communication. For organizations that already provide home-delivered meals or congregate nutrition services, such platforms may help maintain social contact and service continuity for some homebound or geographically isolated older adults (48, 127). These digital spaces enable older adults to connect with peers, learn new skills, and remain engaged, even when homebound or isolated due to physical or geographic constraints.

Public-private partnerships (PPPs) have become a strategic mechanism for tackling complex issues like FI in aging populations. These collaborations, typically between government agencies and private or non-profit organizations, pool resources, expertise, and infrastructure to expand service delivery and reach underserved populations (128, 129). PPPs may expand service capacity and logistical reach, but their effects on quality, cost, equity, and sustainability require formal evaluation (116). One major federal initiative is the U.S. Department of Health and Human Services’ “Food Is Medicine” (FIM) program, which has established four major PPPs to integrate nutrition into the healthcare system (130). Among them, a key partnership with Instacart leverages the company’s technology, logistics network, and research to expand food access for vulnerable populations. Instacart’s Senior Support Service allows adults over 60 to order groceries by phone, addressing both access and digital literacy barriers (130, 131).

Several other local innovations demonstrate the diverse ways technology is being leveraged. In Louisville, KY, a Virtual Supermarket initiative enables older adults and individuals with disabilities to order groceries online from community hubs like libraries and senior housing. By partnering with Amazon Fresh and Kroger to accept SNAP, the program addresses digital and transportation barriers while utilizing trained “Neighbor Food Advocates” to support users in adopting the technology and building food empowerment through education and community organizing (132). In Ohio, the Senior Farmers Market Nutrition Program digitized its traditional voucher system through the Homegrown Benefits mobile app, streamlining transactions, supporting daily reporting, and improving access across diverse user groups, including the Plain Community. Broad training and intuitive design were key to ensuring equitable adoption (133). Similarly, the National Council on Aging’s BenefitsCheckUp tool offers a centralized platform for older adults to identify and apply for eligible food assistance programs, simplifying access to critical services (134). These case examples illustrate the multifaceted and scalable nature of community-based technological interventions. By integrating GIS mapping, mobile food systems, virtual education, and public-private collaboration, communities can more effectively combat FI and promote healthy aging in place.

## 6 Challenges and ethical considerations

As technology becomes more deeply integrated into community-based nutrition interventions for older adults, it is critical to recognize and address the ethical, infrastructural, and social challenges that may arise (135, 136). Without thoughtful planning and inclusive design, these innovations risk reinforcing, rather than reducing, existing health disparities among older populations (137). Three core areas of concern include the digital divide, data privacy and cybersecurity, and the potential for exacerbating inequality in care access.

These challenges are not distributed evenly across settings. The feasibility of technology-supported nutrition interventions depends on local infrastructure, including broadband coverage, device affordability, digital literacy, delivery networks, transportation systems, food assistance programs, and the availability of trained health and social care workers. As a result, an intervention that is feasible in a digitally resourced urban health system may be difficult to implement in rural, low-resource, or fragmented care settings. Transferability should therefore be assessed at the level of local systems rather than assumed from evidence generated in different countries or service environments (138, 139).

One of the most pressing barriers to the successful implementation of digital health and nutrition programs for older adults is the persistent digital divide, a multifaceted disparity involving access to hardware, internet connectivity, digital literacy, and comfort with technology (140). Older adults consistently report more barriers to mobile technology use than younger populations, including difficulty operating devices, mistrust of technology, and lack of confidence in their ability to use it effectively (127). These issues are further compounded by lower rates of both computer use and health-related internet activity among older populations, especially when compared to younger adults (137).

Specific challenges arise from the aging process itself. Sensory impairments such as diminished smell and taste may reduce appetite and complicate dietary tracking, while cognitive decline, including memory loss, can impair the ability to recall food intake or use nutrition apps consistently (19). Functional limitations such as reduced mobility or dexterity may make it difficult to use smartphones or wearables, while environmental changes, such as relocation to assisted living or reliance on caregivers for food preparation, can affect an older adult’s autonomy in managing their nutrition (19). Research by Bailey et al. (87) revealed that older adults with lower health literacy are significantly less likely to own smartphones or use the internet for health-related purposes. Similarly, Levine et al. (141) observed low but gradually increasing adoption of digital health tools among individuals aged 75 and older, highlighting both progress and persistent gaps. Despite the growing number of digital health apps available, there remains a lack of robust evidence linking sociodemographic, behavioral, and medical factors, such as chronic illness, health-related quality of life, or frequency of internet use, to health app adoption and outcomes in this demographic (142).

Encouragingly, recent studies indicate that the internet access gap has narrowed considerably over the past two decades, offering cautious optimism for increased telehealth equity (143). However, digital literacy remains uneven, and efforts to bridge this divide must include not only technology provision and broadband expansion but also user-centered training and support, especially for older adults who may be new to or apprehensive about using digital devices. Moreover, while interventions often focus on equipping patients, the readiness and training of medical professionals to deliver inclusive telehealth and mHealth services must also be prioritized to ensure equitable care delivery (144).

As digital interventions expand into older adults’ health and nutrition, concerns surrounding data privacy, informed consent, and cybersecurity have become increasingly urgent. Older adults with chronic conditions often face challenges such as impaired cognition, visual or auditory impairments, and a general mistrust of digital systems, all of which can hinder informed consent and limit meaningful participation in tech-based interventions (110, 145).

Privacy is a particularly sensitive issue, as many older adults are unfamiliar with how their data may be collected, stored, shared, or used. This generation, having grown up without pervasive digital technology, may struggle more than younger users to understand or trust automated health systems and digital data exchange (146). While efforts to simplify user interfaces and enhance transparency in app design are underway, these have not always kept pace with the rapid development and deployment of new tools. Cybersecurity risks, including data breaches and unauthorized access to sensitive health information, present another layer of complexity, particularly for populations who may not be familiar with how to protect themselves online or even recognize potential threats (89).

To foster trust and ensure ethical implementation, digital nutrition and health technologies must incorporate age-appropriate privacy settings, transparent data usage disclosures, and simplified consent processes (147). Education about data protection, clear opt-in procedures, and responsive support systems are crucial to empowering older adults to safely engage with digital health tools.

Perhaps the greatest ethical challenge lies in the risk of reinforcing or deepening existing health disparities if technological solutions are implemented without deliberate equity-focused design and policy safeguards (148). Older adults with limited income, lower education levels, or marginalized ethnic backgrounds are already more likely to face FI and poorer health outcomes, and they are often the least likely to have access to digital health solutions or the resources needed to engage with them (149, 150). Unless technologies are co-designed with diverse older populations, and unless systems are put in place to subsidize access, support low-literacy users, and ensure cultural relevance, these tools may disproportionately benefit more affluent, digitally connected individuals, leaving the most vulnerable even further behind (149).

**Figure 1:**
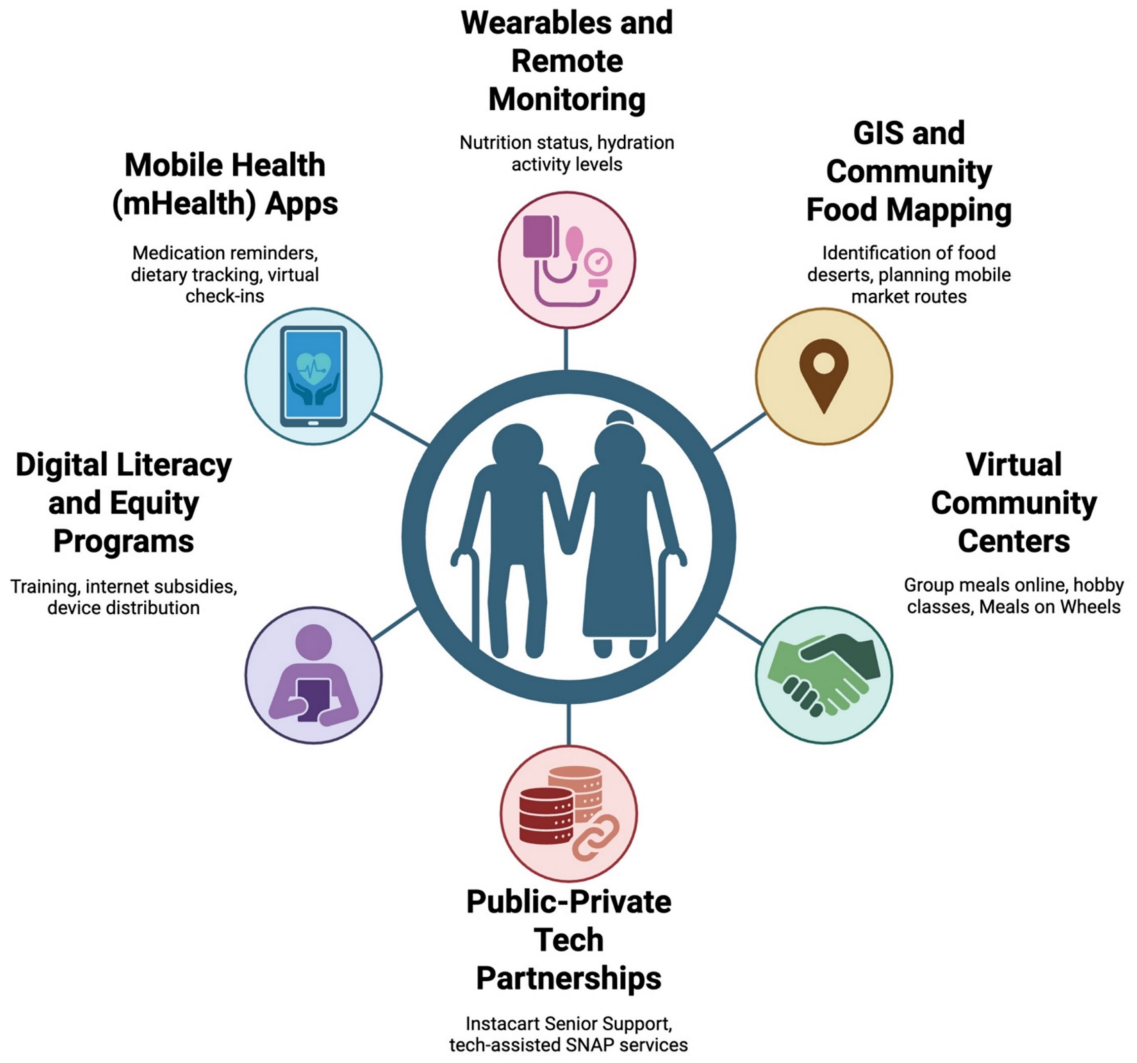
Integrated technology-enabled strategies addressing food insecurity, nutrition access, and social isolation among older adults. The figure summarizes complementary digital, community-based, and health-system strategies that may support older adults experiencing or at risk of food insecurity. Mobile health (mHealth) applications may assist with dietary tracking, medication reminders, meal planning, grocery ordering, and virtual check-ins, particularly when combined with caregiver or professional support. Wearables and remote monitoring tools may provide information on nutrition-relevant indicators such as physical activity, hydration-related parameters, glucose monitoring, and other health metrics, although their usefulness depends on clinical validation, user training, and clear response pathways. Geographic information systems (GIS) and community food mapping can help identify food deserts, characterize geographic barriers to healthy food access, and guide the placement of mobile markets, delivery routes, and community nutrition resources. Virtual community centers and digitally supported congregate or home-delivered meal programs may help maintain social contact, provide nutrition education, and support shared food practices among homebound or socially isolated older adults. Public–private technology partnerships may expand food access through grocery delivery platforms, benefit-navigation tools, and technology-assisted food assistance enrollment, including services linked to the Supplemental Nutrition Assistance Program (SNAP). Digital literacy and equity programs, including device distribution, internet subsidies, training, and accessible design, are essential to prevent technology-based interventions from widening existing disparities. These strategies should be understood as adjuncts to, rather than replacements for, adequate food assistance, caregiver support, community services, and equitable health and social care systems (19, 60, 67, 137–139, 144). GIS, geographic information systems; mHealth, mobile health; SNAP, supplemental nutrition assistance program; PPP, public–private partnership.

Finally, the promise of digital health and nutrition support for older adults is substantial, but so too are the risks if implementation lacks ethical rigor, user inclusivity, and structural support. Bridging the digital divide, safeguarding user data, and proactively designing against inequality are not peripheral concerns; they are central to realizing technology’s full potential to promote health equity in aging populations. These digital solutions, when implemented cohesively, demonstrate a promising framework for addressing both nutritional and social needs of older adults in community settings (Figure 1).

## 7 Future directions: building inclusive and scalable solutions

As digital health technologies advance, older adults should not be treated as a homogeneous group or as passive recipients of innovation. Older adults’ intention to adopt digital technologies is shaped by multiple interacting factors, including health status, motivation, perceived usefulness, social influence, confidence, and functional features of the technology (151). Some older adults are active and skilled users of everyday technologies, adapting devices and platforms to fit their routines, preferences, and care arrangements (87, 142). Others may have limited access, low confidence, sensory or cognitive barriers, concerns about privacy, or a clear preference for non-digital forms of support. Future technology-supported nutrition and food access interventions should therefore be designed around heterogeneity rather than age alone, recognizing that non-use may reflect exclusion, but may also represent a valid preference (67, 151–153).

However, many current digital health tools lack age-appropriate features, often failing to account for common challenges like reduced vision or hearing, limited dexterity, and less familiarity with smartphones. Future solutions must embrace tailored universal design principles, incorporating features like voice commands, adjustable font sizes, simplified navigation, and offline functionality to enhance usability across diverse abilities. It is also vital to include older adults as co-designers, testers, and advisors in all development phases to ensure interventions meet real-world needs and build trust.

Beyond technical design, scalability depends on the capacity of health, social care, and community systems to integrate digital tools into existing workflows without displacing essential human support. Combining mobile health tools with in-person follow-ups, telephone outreach, caregiver communication, or electronic health records may improve continuity and personalization of care in well-resourced settings, but these approaches are not universally transferable (154, 155). Their feasibility depends on broadband coverage, device affordability, reimbursement mechanisms, data interoperability, workforce capacity, technical support, and the availability of trusted community organizations. In lower-resource settings, or in regions with fragmented health information systems, lower digital literacy, or limited social care infrastructure, simpler hybrid models, such as telephone-supported grocery ordering, community hubs, mobile markets, or digitally assisted benefit navigation, may be more realistic than fully integrated app- or EHR-based systems. Future implementation should therefore be context-specific, allowing technology to strengthen existing food and care systems rather than substituting for underfunded services.

### 7.1 Limitations of the review

This review has several limitations. Because it was designed as a narrative review, it did not use systematic screening, formal risk-of-bias assessment, or meta-analysis. The findings should therefore be interpreted as a critical synthesis rather than a comprehensive estimate of intervention effectiveness. The available literature on technology-supported nutrition access for older adults is geographically uneven. Many of the intervention studies, digital health examples, and policy initiatives discussed in this review originate from high-income and Western settings, particularly contexts with relatively developed health information systems, broadband infrastructure, and formal food assistance programs. Evidence from non-Western and low- and middle-income settings remains more limited, especially for technology-supported interventions specifically targeting food-insecure older adults. Many included studies address related populations or outcomes rather than directly testing food security and social isolation outcomes in older adults. For this reason, the review distinguishes direct evidence from indirect or contextual evidence and avoids treating findings as universally generalizable. Future research should include more studies from diverse geographic and income settings and should evaluate implementation feasibility, acceptability, equity, and sustainability alongside nutritional and social outcomes.

## 8 Conclusion

As the global population continues to age, addressing the complex intersection of FI, nutrition, and social isolation among older adults has become an urgent public health priority. These challenges are connected through shared structural and interpersonal pathways, including poverty, mobility limitations, reduced access to transportation, chronic illness, loss of shared meals, and limited social support for shopping, cooking, and navigation food assistance. Emerging technologies, including wearable devices, telehealth platforms, community-based digital initiatives, and mobile health applications, offer promising pathways to support older adults in maintaining nutritional well-being and social connection. However, realizing the full potential of these innovations requires deliberate design, inclusive strategies, and supportive policy frameworks that center older adults not merely as recipients of technology, but as active participants in its development and use.

Wearable technologies and remote monitoring tools may contribute to earlier recognition of nutritional risk when combined with clinical assessment, caregiver input, and appropriate referral pathways. Community-based approaches such as GIS mapping, mobile markets, and virtual community centers may help reduce selected geographic, informational and social barriers to food access, particularly when supported by local organizations and policy infrastructure. However, the evidence base remains heterogeneous, and many interventions have been evaluated in specific populations, short-term settings, or high-resource contexts. These strategies should therefore be understood as complementary tools that may support nutrition access, social connection, and autonomy when implemented with adequate human support, rather than as universally effective solutions.

Substantial challenges remain. The digital divide, driven by disparities in access to devices, internet connectivity, and digital literacy, continues to limit the reach of these solutions among vulnerable subgroups. Ethical concerns around data privacy, consent, and cybersecurity are particularly salient for older adults, who may face cognitive or sensory limitations and harbor legitimate distrust toward unfamiliar technologies. Without careful attention to equity, accessibility, and user empowerment, there is a risk that digital innovations may inadvertently exacerbate existing inequalities.

Looking forward, future efforts must prioritize inclusive and scalable technology development that addresses the diverse needs of older adults. This includes adopting universal design principles, embedding digital tools within interdisciplinary care ecosystems, and supporting robust infrastructure for digital literacy training and ongoing support. Equally critical is ensuring that older adults are actively involved in the design, testing, and evaluation of these tools. Ultimately, while technology alone cannot resolve FI or social isolation, it can be a powerful adjunct when implemented with intentionality, empathy, and collaboration. As healthcare systems, researchers, and policymakers confront the realities of aging societies, it is imperative to embrace technology not just as a tool of convenience, but as a vehicle for dignity, equity, and meaningful connection in the lives of older adults. Importantly, while digital health technologies and telemedicine can enhance access to healthcare services and support patient management, they should not be regarded as a replacement for professional medical oversight. The monitoring and interpretation of health-related information should be conducted under the guidance of qualified healthcare professionals to ensure accuracy, safety and appropriate clinical decision-making. Therefore, the integration of digital health solutions into healthcare systems should be accompanied by continuous professional supervision and patient support.

## References

[1] Howe-Burris M Giroux S Waldman K DeBruicker Valliant J Babb A Czebotar K . The interactions of food security, health, and loneliness among rural older adults before and after the onset of COVID-19. Nutrients. (2022) 14:5076. doi: 10.3390/nu14235076, 36501106 PMC9739434

[2] Arzhang P Abbasi SH Sarsangi P Malekahmadi M Nikbaf-Shandiz M Bellissimo N . Prevalence of household food insecurity among a healthy Iranian population: a systematic review and meta-analysis. Front Nutr. (2022) 9:9. doi: 10.3389/fnut.2022.1006543, 36458165 PMC9707736

[3] FAO, IFAD, UNICEF, WFP, WHO. "The state of food security and nutrition in the world 2020". In: The State of Food Security and Nutrition in the World 2020. Rome, Italy: Food and Agriculture Organization of the United Nations (FAO) (2020). doi: 10.4060/ca9692en

[4] Pereira MHQ Pereira MLAS Teles BKA dos Santos Pereira DB De Campos GC del Carmen Bisi Molina M. Food insecurity and malnutrition in older adults from the family health strategy in the northeast of Brazil. Arch Latinoam Nutr. (2022) 72:274–84. doi: 10.37527/2022.72.4.005

[5] Lee MJ Almidani L Samuel L Swenor BK Ehrlich JR Varadaraj V. Vision impairment and food insecurity in the national health and aging trends study. Front Epidemiol. (2024) 4:1353083. doi: 10.3389/fepid.2024.1353083, 38751732 PMC11094228

[6] Xiao Y Yin S Bai Y Wang J Cui J Yang Y . A positive association between food insecurity and the prevalence of overactive bladder in U.S. adults. Front Nutr. (2024) 10:10. doi: 10.3389/fnut.2023.1329687, 38370980 PMC10870421

[7] Zahidi F Khalid M Surkan PJ Azadbakht L. Associations between food insecurity and common mental health problems among reproductive-aged women in Kabul-Afghanistan. Front Nutr. (2022) 8:8. doi: 10.3389/fnut.2021.794607, 35047547 PMC8761756

[8] Lee JS. Food insecurity in older adults. J Nutr. (2022) 152:1808–9. doi: 10.1093/jn/nxac112, 35732466

[9] Pooler JA Hartline-Grafton H DeBor M Sudore RL Seligman HK. Food insecurity: a key social determinant of health for older adults. J Am Geriatr Soc. (2019) 67:421–4. doi: 10.1111/jgs.15736, 30586154 PMC6816803

[10] Ziliak JP Gundersen C The Health Consequences of Senior Hunger in the United States: Evidence from the 1999–2016 NHANES. (2021) Chicago, IL: Feeding America.

[11] Vaudin AM Moshfegh AJ Sahyoun NR. Measuring food insecurity in older adults using both physical and economic food access, NHANES 2013-18. J Nutr. (2022) 152:1953–62. doi: 10.1093/jn/nxac058, 35285903

[12] Gujo MM Modiba LM. Food insecurity and its determinants in pastoralist and agrarian communities in south Omo zone, southern Ethiopia: a community-based cross-sectional study. Front Public Health. (2024) 12:1482208. doi: 10.3389/fpubh.2024.1482208, 39463896 PMC11505120

[13] Locher JL Ritchie CS Roth DL Sen B Vickers Douglas K Vailas LI . Food choice among homebound older adults: motivations and perceived barriers NIH public access. J Nutr Health Aging. (2009) 13:659–64. doi: 10.1007/s12603-009-0194-7 19657547 PMC2749957

[14] Coleman-Jensen A. Rabbitt M. P. Gregory C. A. Singh A. Household Food Security in the United States in 2020. Washington, DC: U.S. Department of Agriculture, Economic Research Service. (2021). Available online at: www.ers.usda.gov (Accessed February 25, 2026).

[15] Jih J Stijacic-Cenzer I Seligman HK Boscardin WJ Nguyen TT Ritchie CS. Chronic disease burden predicts food insecurity among older adults. Public Health Nutr. (2018) 21:1737–42. doi: 10.1017/S1368980017004062, 29388533 PMC6204426

[16] Burris M Kihlstrom L Arce KS Prendergast K Dobbins J McGrath E . Food insecurity, loneliness, and social support among older adults. J Hunger Environ Nutr. (2021) 16:29–44. doi: 10.1080/19320248.2019.1595253

[17] Hanna KL Collins PF. Relationship between living alone and food and nutrient intake. Nutr Rev. (2015) 73:594–611. doi: 10.1093/nutrit/nuv024, 26269488

[18] Nicholson NR. A review of social isolation: An important but underassessed condition in older adults. J Prim Prev. (2012) 33:137–52. doi: 10.1007/s10935-012-0271-2, 22766606

[19] Takemoto M Manini TM Rosenberg DE Lazar A Zlatar ZZ Das SK . Diet and activity assessments and interventions using technology in older adults. Am J Prev Med. (2018) 55:e105–15. doi: 10.1016/j.amepre.2018.06.005, 30241621 PMC7176031

[20] Astell AJ Hwang F Brown LJE Timon C Maclean LM Smith T . Validation of the NANA (novel assessment of nutrition and ageing) touch screen system for use at home by older adults. Exp Gerontol. (2014) 60:100–7. doi: 10.1016/j.exger.2014.10.008, 25456843

[21] Baethge C Goldbeck-Wood S Mertens S. SANRA—a scale for the quality assessment of narrative review articles. Res Integr Peer Rev. (2019) 4:5. doi: 10.1186/s41073-019-0064-8, 30962953 PMC6434870

[22] Leung CW Wolfson JA. Food insecurity among older adults: 10-year National Trends and associations with diet quality. J Am Geriatr Soc. (2021) 69:964–71. doi: 10.1111/jgs.16971, 33403662 PMC8341441

[23] World Health Organization. Ageing and health. (2024). Available online at: https://www.who.int/news-room/fact-sheets/detail/ageing-and-health (Accessed February 25, 2026).

[24] Fong JH. Disability incidence and functional decline among older adults with major chronic diseases. BMC Geriatr. (2019) 19:323. doi: 10.1186/s12877-019-1348-z, 31752701 PMC6873710

[25] Lock S. Baumgart M. Whiting C. G. McGuire L. Iskander J. Thorpe P. Healthy Aging: Promoting well-Being in older Adults. Atlanta, GA: Centers for Disease Control and Prevention (CDC), Public Health Grand Rounds. (2017).

[26] Barrea L Vetrani C Verde L Frias-Toral E Ceriani F Cernea S . Comprehensive approach to medical nutrition therapy in patients with type 2 diabetes mellitus: from diet to bioactive compounds. Antioxidants. (2023) 12:12. doi: 10.3390/antiox12040904, 37107279 PMC10135374

[27] Blake CE Frongillo EA Warren AM Constantinides SV Rampalli KK Bhandari S. Elaborating the science of food choice for rapidly changing food systems in low-and middle-income countries. Glob Food Sec. (2021) 28:100503. doi: 10.1016/j.gfs.2021.100503, 38826717

[28] Himmelgreen D Romero-Daza N Heuer J Lucas W Salinas-Miranda AA Stoddard T. Using syndemic theory to understand food insecurity and diet-related chronic diseases. Soc Sci Med. (2022) 295:113124. doi: 10.1016/j.socscimed.2020.113124, 32586635

[29] Schwartz N Buliung R Wilson K. Disability and food access and insecurity: a scoping review of the literature. Health Place. (2019) 57:107–21. doi: 10.1016/j.healthplace.2019.03.011 31026771

[30] Engel JH Siewerdt F Jackson R Akobundu U Wait C Sahyoun N. Hardiness, depression, and emotional well-being and their association with appetite in older adults. J Am Geriatr Soc. (2011) 59:482–7. doi: 10.1111/j.1532-5415.2010.03274.x, 21391938

[31] Osei-Owusu C Dhillon S Luginaah I. The impact of food insecurity on mental health among older adults residing in low- and middle-income countries: a systematic review. PLoS One. (2024) 19:e0301046–22. doi: 10.1371/journal.pone.0301046, 38530847 PMC10965102

[32] Homenko DR Morin PC Eimicke JP Teresi JA Weinstock RS. Food insecurity and food choices in rural older adults with diabetes receiving nutrition education via telemedicine. J Nutr Educ Behav. (2010) 42:404–9. doi: 10.1016/j.jneb.2009.08.001, 21070978

[33] Janda KM Ranjit N Salvo D Hoelscher DM Nielsen A Casnovsky J . Examining geographic food access, food insecurity, and Urbanicity among diverse, low-income participants in Austin, Texas. Int J Environ Res Public Health. (2022) 19:5108. doi: 10.3390/ijerph19095108, 35564504 PMC9104388

[34] Piontak JR Schulman MD. Food insecurity in rural America. Contexts. (2014) 13:75–7. doi: 10.1177/1536504214545766

[35] Smith L. The geography and causes of food insecurity in developing countries. Agric Econ. (2000) 22:199–215. doi: 10.1016/S0169-5150(99)00051-1

[36] De Marchis EH Torres JM Fichtenberg C Gottlieb LM. Identifying food insecurity in health care settings. Fam Community Health. (2019) 42:20–9. doi: 10.1097/FCH.0000000000000208 30431466

[37] Uscher-Pines L McCullough CM Sousa JL Lee SD Ober AJ Camacho D . Changes in in-person, audio-only, and video visits in California’s federally qualified health centers, 2019-2022. JAMA. (2019) 329:1219. doi: 10.1001/jama.2023.1307, 37039799 PMC10091174

[38] Fernandes SG Rodrigues AM Nunes C Santos O Gregório MJ de Sousa RD . Food insecurity in older adults: results from the epidemiology of chronic diseases cohort study 3. Front Med. (2018) 5:203. doi: 10.3389/fmed.2018.00203, 30050904 PMC6052142

[39] Aljahdali AA Na M Leung CW. Food insecurity and health-related quality of life among a nationally representative sample of older adults: cross-sectional analysis. BMC Geriatr. (2024) 24:126. doi: 10.1186/s12877-024-04716-9, 38302907 PMC10835917

[40] Bergmans RS Mezuk B Zivin K. Food insecurity and geriatric hospitalization. Int J Environ Res Public Health. (2019) 16:2294. doi: 10.3390/ijerph16132294, 31261648 PMC6651817

[41] Reytor-González C Simancas-Racines D Román-Galeano NM Campuzano-Donoso M Carella AM Zambrano-Villacres R . Obesity and breast cancer: exploring the nexus of chronic inflammation, metabolic dysregulation, and nutritional strategies. Food Agric Immunol. (2025) 36:36. doi: 10.1080/09540105.2025.2521270, 37339054

[42] Reytor-González C Simancas-Racines D Jiménez-Flores E Campuzano-Donoso M Carella AM Coppola L . Oesophageal adenocarcinoma, obesity, and cancer: the role of nutrition in prevention and management. Food Agric Immunol. (2025) 36:36. doi: 10.1080/09540105.2025.2510951, 37339054

[43] Reytor-González C Parise-Vasco JM González N Simancas-Racines A Zambrano-Villacres R Zambrano AK . Obesity and periodontitis: a comprehensive review of their interconnected pathophysiology and clinical implications. Front Nutr. (2024) 11:11. doi: 10.3389/fnut.2024.1440216, 39171112 PMC11335523

[44] Sarno G Reytor-González C Frías-Toral E Campuzano-Donoso M Katsanos CS Simancas-Racines D. Navigating the weight: the impact of obesity on gastrointestinal Cancer surgery and strategies for improved outcomes. Semin Cancer Biol. (2025) 114:138–49. doi: 10.1016/j.semcancer.2025.06.010, 40581314

[45] Simancas-Racines D Reytor-González C Frias-Toral E Katsanos CS Hidalgo R. Weighty matters: unraveling the impact of obesity on colorectal cancer and nutritional interventions. Semin Cancer Biol. (2025) 114:29–40. doi: 10.1016/j.semcancer.2025.06.004, 40482846

[46] Eccles A. Remote care technologies, older people and the social care crisis in the United Kingdom: a multiple streams approach to understanding the ‘silver bullet’ of telecare policy. Ageing Soc. (2021) 41:1726–47. doi: 10.1017/S0144686X19001776

[47] Hamblin KA. Technology in care systems: displacing, reshaping, reinstating or degrading roles? New Technol Work Employ. (2022) 37:41–58. doi: 10.1111/ntwe.12229, 35911255 PMC9304303

[48] Dickinson A Wills W. Meals on wheels services and the food security of older people. Health Soc Care Community. (2022) 30:30. doi: 10.1111/hsc.14092, 36300541 PMC10092458

[49] Valliant JCD Burris ME Czebotar K Stafford PB Giroux SA Babb A . Navigating food insecurity as a rural older adult: the importance of congregate meal sites, social networks and transportation services. J Hunger Environ Nutr. (2022) 17:593–614. doi: 10.1080/19320248.2021.1977208

[50] Sen K Kruse CS Mileski M Ramamonjiarivelo Z. Interventions to reduce social isolation and food insecurity in older adults: a systematic review. Front Nutr. (2025) 12:12. doi: 10.3389/fnut.2025.1607057, 40547373 PMC12178855

[51] Smith AC Thomas E Snoswell CL Haydon H Mehrotra A Clemensen J . Telehealth for global emergencies: implications for coronavirus disease 2019 (COVID-19). J Telemed Telecare. (2020) 26:309–13. doi: 10.1177/1357633X20916567, 32196391 PMC7140977

[52] Rodríguez Veintimilla D Noles Rodríguez LM Frias-Toral E. La pandemia por COVID-19: hambre, malnutrición y consecuencias sociales para América Latina y el Caribe. Rev Nutr Clin Metab. (2022) 5:4–5. doi: 10.35454/rncm.v5n1.374

[53] Shaver J. The state of telehealth before and after the COVID-19 pandemic. Prim Care. (2022) 49:517–30. doi: 10.1016/j.pop.2022.04.002, 36357058 PMC9035352

[54] Contreras CM Metzger GA Beane JD Dedhia PH Ejaz A Pawlik TM. Telemedicine: patient-provider clinical engagement during the COVID-19 pandemic and beyond. J Gastrointest Surg. (2020) 24:1692–7. doi: 10.1007/s11605-020-04623-5, 32385614 PMC7206900

[55] Losorelli SD Vendra V Hildrew DM Woodson EA Brenner MJ Sirjani DB. The future of telemedicine: revolutionizing health care or flash in the pan? Otolaryngol Head Neck Surg. (2021) 165:239–43. doi: 10.1177/0194599820983330 33399500

[56] Grossman SN Han SC Balcer LJ Kurzweil A Weinberg H Galetta SL . Rapid implementation of virtual neurology in response to the COVID-19 pandemic. Neurology. (2020) 94:1077–87. doi: 10.1212/WNL.0000000000009677, 32358217

[57] Prasad A Brewster R Newman JG Rajasekaran K. Optimizing your telemedicine visit during the COVID −19 pandemic: practice guidelines for patients with head and neck cancer. Head Neck. (2020) 42:1317–21. doi: 10.1002/hed.26197, 32343458 PMC7267295

[58] Saleem SM Pasquale LR Sidoti PA Tsai JC. Virtual ophthalmology: telemedicine in a COVID-19 era. Am J Ophthalmol. (2020) 216:237–42. doi: 10.1016/j.ajo.2020.04.029, 32360862 PMC7191296

[59] Muscogiuri G Verde L Sulu C Katsiki N Hassapidou M Frias-Toral E . Mediterranean diet and obesity-related disorders: what is the evidence? Curr Obes Rep. (2022) 11:287–304. doi: 10.1007/s13679-022-00481-1, 36178601 PMC9729142

[60] Sun C Sun L Xi S Zhang H Wang H Feng Y . Mobile phone–based telemedicine practice in older Chinese patients with type 2 diabetes mellitus: randomized controlled trial. JMIR Mhealth Uhealth. (2019) 7:e10664. doi: 10.2196/10664, 30609983 PMC6682265

[61] Calton B Abedini N Fratkin M. Telemedicine in the time of coronavirus. J Pain Symptom Manag. (2020) 60:e12–4. doi: 10.1016/j.jpainsymman.2020.03.019, 32240756 PMC7271287

[62] Reytor-González C Annunziata G Campuzano-Donoso M Morales-López T Basantes-Tituaña C Fascì-Spurio F . Endocrinologist’s crucial role in metabolic dysfunction-associated steatotic liver disease: a comprehensive review. Minerva Endocrinol. (2025) 50:209–26. doi: 10.23736/S2724-6507.24.04314-8 40116171

[63] Reytor-González C Simancas-Racines D Campuzano-Donoso M Castano Jimenez J Román-Galeano NM Sarno G . Harnessing nutrition to combat MASLD: a comprehensive guide to food-based therapeutic strategies. Food Agric Immunol. (2025) 36:36. doi: 10.1080/09540105.2025.2496499, 37339054

[64] Kaufman-Shriqui V Sherf-Dagan S Boaz M Birk R. Virtual nutrition consultation: what can we learn from the COVID-19 pandemic? Public Health Nutr. (2021) 24:1166–73. doi: 10.1017/S1368980021000148, 33436134 PMC7870906

[65] Daud NLHM Nor NM Jaafar NH Bakar WAMA Shukri NAM. The feasibility and effectiveness of telenutrition for remote dietary consultation: a systematic review and meta-analysis. Curr Res Nutr Food Sci. (2025) 13:46–60. doi: 10.12944/CRNFSJ.13.1.3

[66] Calcaterra V Verduci E Vandoni M Rossi V Di Profio E Carnevale Pellino V . Telehealth: a useful tool for the management of nutrition and exercise programs in pediatric obesity in the COVID-19 era. Nutrients. (2021) 13:3689. doi: 10.3390/nu13113689, 34835945 PMC8618189

[67] Awan M Ali S Ali M Abrar MF Ullah H Khan D. Usability barriers for elderly users in smartphone app usage: an analytical hierarchical process-based prioritization. Sci Program. (2021) 2021:1–14. doi: 10.1155/2021/2780257

[68] Tomé-Carneiro J Visioli F Espinosa-Salinas I López-Legarrea P Martínez JA. Nutrient profile systems: a review of applications, validation and emerging developments. Int J Food Sci Nutr. (2026) 77:110–25. doi: 10.1080/09637486.2026.2629989, 41725069

[69] Abeltino A Riente A Bianchetti G Serantoni C De Spirito M Capezzone S . Digital applications for diet monitoring, planning, and precision nutrition for citizens and professionals: a state of the art. Nutr Rev. (2025) 83:e574–601. doi: 10.1093/nutrit/nuae035, 38722240 PMC11986332

[70] Milne-Ives M Lam C De Cock C Van Velthoven MH Meinert E. Mobile apps for health behavior change in physical activity, diet, drug and alcohol use, and mental health: systematic review. JMIR Mhealth Uhealth. (2020) 8:e17046. doi: 10.2196/17046, 32186518 PMC7113799

[71] Ha SK Lee HS Park HY. Twelve smartphone applications for health management of older adults during the covid-19 pandemic. Int J Environ Res Public Health. (2021) 18:18. doi: 10.3390/ijerph181910235, 34639536 PMC8507820

[72] Trifan A Oliveira M Oliveira JL. Passive sensing of health outcomes through smartphones: systematic review of current solutions and possible limitations. JMIR Mhealth Uhealth. (2019) 7:e12649. doi: 10.2196/12649, 31444874 PMC6729117

[73] Facon M Skura BJ Nakai S. Potential for immunological supplementation of foods. Food Agric Immunol. (1993) 5:85–91. doi: 10.1080/09540109309354787

[74] Mummah S Robinson TN Mathur M Farzinkhou S Sutton S Gardner CD. Effect of a mobile app intervention on vegetable consumption in overweight adults: a randomized controlled trial. Int J Behav Nutr Phys Act. (2017) 14:125. doi: 10.1186/s12966-017-0563-2, 28915825 PMC5603006

[75] Clarke P Evans SH Neffa-Creech D. Mobile app increases vegetable-based preparations by low-income household cooks: a randomized controlled trial. Public Health Nutr. (2019) 22:714–25. doi: 10.1017/S1368980018003117, 30472970 PMC6521788

[76] Blackburne T Rodriguez A Johnstone SJ. A serious game to increase healthy food consumption in overweight or obese adults: randomized controlled trial. JMIR Serious Games. (2016) 4:e10. doi: 10.2196/games.5708, 27417192 PMC4963607

[77] Garcia-Ortiz L Recio-Rodriguez JI Agudo-Conde C Patino-Alonso MC Maderuelo-Fernandez J-A Repiso Gento I . Long-term effectiveness of a smartphone app for improving healthy lifestyles in general population in primary care: randomized controlled trial (Evident II study). JMIR Mhealth Uhealth. (2018) 6:e107. doi: 10.2196/mhealth.9218, 29702473 PMC5948409

[78] Bakre S Shea B Ortega K Scharen J Langheier J Hu E. Changes in food insecurity among individuals using a telehealth and nutrition platform: longitudinal study. JMIR Form Res. (2022) 6:e41418. doi: 10.2196/41418, 36282563 PMC9644245

[79] Biasini B Tiboni-Oschilewski O Monica E Deon V Rapetti V Scazzina F . Evaluation of the potential of promoting healthy and sustainable food choices in a worksite canteen through an app-based intervention. Int J Food Sci Nutr. (2025) 76:634–45. doi: 10.1080/09637486.2025.2543242, 40774165

[80] Tandon A Kaur P Bhatt Y Mäntymäki M Dhir A. Why do people purchase from food delivery apps? A consumer value perspective. J Retail Consum Serv. (2021) 63:102667. doi: 10.1016/j.jretconser.2021.102667

[81] Ray A Dhir A Bala PK Kaur P. Why do people use food delivery apps (FDA)? A uses and gratification theory perspective. J Retail Consum Serv. (2019) 51:221–30. doi: 10.1016/j.jretconser.2019.05.025

[82] Hwang S Johnson CM Charles J Biediger-Friedman L. Food delivery apps and their potential to address food insecurity in older adults: a review. Int J Environ Res Public Health. (2024) 21:1197. doi: 10.3390/ijerph21091197, 39338080 PMC11431773

[83] Wang X Zhao F Tian X Min S von Cramon-Taubadel S Huang J . How online food delivery platforms contributed to the resilience of the urban food system in China during the COVID-19 pandemic. Glob Food Sec. (2022) 35:100658. doi: 10.1016/j.gfs.2022.100658, 36248772 PMC9554343

[84] Seligman HK Bindman AB Vittinghoff E Kanaya AM Kushel MB. Food insecurity is associated with diabetes mellitus: results from the National Health Examination and nutrition examination survey (NHANES) 1999–2002. J Gen Intern Med. (2007) 22:1018–23. doi: 10.1007/s11606-007-0192-6, 17436030 PMC2583797

[85] Lunde P Nilsson BB Bergland A Kværner KJ Bye A. The effectiveness of smartphone apps for lifestyle improvement in noncommunicable diseases: systematic review and meta-analyses. J Med Internet Res. (2018) 20:e162. doi: 10.2196/jmir.9751, 29728346 PMC5960039

[86] Dounavi K Tsoumani O. Mobile health applications in weight management: a systematic literature review. Am J Prev Med. (2019) 56:894–903. doi: 10.1016/j.amepre.2018.12.005, 31003801

[87] Bailey SC O’Conor R Bojarski EA Mullen R Patzer RE Vicencio D . Literacy disparities in patient access and health-related use of internet and mobile technologies. Health Expect. (2015) 18:3079–87. doi: 10.1111/hex.12294, 25363660 PMC4417455

[88] Van Den Berg N Schumann M Kraft K Hoffmann W. Telemedicine and telecare for older patients - a systematic review. Maturitas. (2012) 73:94–114. doi: 10.1016/j.maturitas.2012.06.010, 22809497

[89] Golshany H Ni Y Yu Q Fan L. IoT-enabled smart kitchen technologies and their impact on food storage, preparation, and culinary experiences: a systematic review. Food Res Int. (2025) 213:116557. doi: 10.1016/j.foodres.2025.116557, 40436590

[90] Almassar KM Khasawneh MT. Using IoT and AI to replenish household food supplies: a systematic review. J Smart Cities Society. (2024) 3:23–62. doi: 10.3233/SCS-230022

[91] Kosch T Woźniak PW Brady E Schmidt A. "Smart kitchens for people with cognitive impairments". In: Proceedings of the 2018 CHI Conference on Human Factors in Computing Systems. New York, NY: ACM (2018). p. 1–12.

[92] Blasco R Marco Á Casas R Cirujano D Picking R. A smart kitchen for ambient assisted living. Sensors. (2014) 14:1629–53. doi: 10.3390/s140101629, 24445412 PMC3926629

[93] Chi P Chen J Chu H Chen B-Y. "Enabling nutrition-aware cooking in a smart kitchen". In: CHI ‘07 Extended Abstracts on Human Factors in Computing Systems. New York, NY: ACM (2007). p. 2333–8.

[94] Simancas-Racines D Campuzano-Donoso M Román-Galeano NM Zambrano-Villacres R Memoli P Verde L . Obesity and endometrial cancer: biological mechanisms, nutritional strategies, and clinical perspectives. Food Agric Immunol. (2025) 36:36. doi: 10.1080/09540105.2025.2510961, 37339054

[95] Kuoppamäki S Tuncer S Eriksson S McMillan D. Designing kitchen technologies for ageing in place. Proc ACM Interact Mob Wearable Ubiquitous Technol. (2021) 5:1–19. doi: 10.1145/3463516

[96] Tricás-Vidal HJ Lucha-López MO Hidalgo-García C Vidal-Peracho MC Monti-Ballano S Tricás-Moreno JM. Health habits and wearable activity tracker devices: analytical cross-sectional study. Sensors. (2022) 22:2960. doi: 10.3390/s22082960, 35458945 PMC9031391

[97] Köhler C Bartschke A Fürstenau D Schaaf T Salgado-Baez E. The value of smartwatches in the health care sector for monitoring, nudging, and predicting: viewpoint on 25 years of research. J Med Internet Res. (2024) 26:e58936. doi: 10.2196/58936, 39356287 PMC11549588

[98] Vaddiraju S Burgess DJ Tomazos I Jain FC Papadimitrakopoulos F. Technologies for Continuous Glucose Monitoring: current problems and future promises. J Diabetes Sci Technol. (2010) 4:1540–62. doi: 10.1177/193229681000400632, 21129353 PMC3005068

[99] Chen X Kamavuako EN. Vision-based methods for food and fluid intake monitoring: a literature review. Sensors. (2023) 23:6137. doi: 10.3390/s23136137, 37447988 PMC10346353

[100] Cappon G Vettoretti M Sparacino G Facchinetti A. Continuous glucose monitoring sensors for diabetes management: a review of technologies and applications. Diabetes Metab J. (2019) 43:383–97. doi: 10.4093/dmj.2019.0121, 31441246 PMC6712232

[101] Miller EM. Using continuous glucose monitoring in clinical practice. Clin Diabetes. (2020) 38:429–38. doi: 10.2337/cd20-0043, 33384468 PMC7755046

[102] Jafleh EA Alnaqbi FA Almaeeni HA Faqeeh S Alzaabi MA Al Zaman K. The role of wearable devices in chronic disease monitoring and patient care: a comprehensive review. Cureus. (2024). doi: 10.7759/cureus.68921, 39381470 PMC11461032

[103] Linseisen J Renner B Gedrich K Wirsam J Holzapfel C Lorkowski S . Data in personalized nutrition: bridging biomedical, psycho-behavioral, and food environment approaches for population-wide impact. Adv Nutr. (2025) 16:100377. doi: 10.1016/j.advnut.2025.100377, 39842719 PMC12281445

[104] Chapela SP Martinuzzi ALN Llobera ND Ceriani F Gonzalez V Montalvan M . Obesity and micronutrients deficit, when and how to suplement. Food Agric Immunol. (2024) 35:35. doi: 10.1080/09540105.2024.2381725, 37339054

[105] Ruiz-Pozo VA Guevara-Ramírez P Paz-Cruz E Tamayo-Trujillo R Cadena-Ullauri S Frias-Toral E . The role of the Mediterranean diet in prediabetes management and prevention: a review of molecular mechanisms and clinical outcomes. Food Agric Immunol. (2024) 35:35. doi: 10.1080/09540105.2024.2398042, 37339054

[106] Sun Y Chen J Ji M Li X. Wearable Technologies for Health Promotion and Disease Prevention in older adults: systematic scoping review and evidence map. J Med Internet Res. (2025) 27:e69077. doi: 10.2196/69077, 40553512 PMC12238792

[107] Pahor M Guralnik JM Ambrosius WT Blair S Bonds DE Church TS . Effect of structured physical activity on prevention of major mobility disability in older adults. JAMA. (2014) 311:2387–96. doi: 10.1001/jama.2014.5616, 24866862 PMC4266388

[108] Amiri M Li J Hasan W. Personalized flexible meal planning for individuals with diet-related health concerns: system design and feasibility validation study. JMIR Form Res. (2023) 7:e46434. doi: 10.2196/46434, 37535413 PMC10436119

[109] Dreier A Rogalski H Oppermann RF Terschüren C Van Den Berg N Hoffmann W. A curriculum for nurses in Germany undertaking medically-delegated tasks in primary care. J Adv Nurs. (2010) 66:635–44. doi: 10.1111/j.1365-2648.2009.05167.x, 20423398

[110] Zaman SB Khan RK Evans RG Thrift AG Maddison R Shariful Islam SM. Exploring barriers to and enablers of the adoption of information and communication technology for the care of older adults with chronic diseases: scoping review. JMIR Aging. (2022). doi: 10.2196/25251, 34994695 PMC8783284

[111] Marshall S Agarwal E Young A Isenring E. Role of domiciliary and family carers in individualised nutrition support for older adults living in the community. Maturitas. (2017):20–9. doi: 10.1016/j.maturitas.2017.01.004, 28274324

[112] Suresh A Shobna Salaria M Morya S Khalid W Afzal FA . Dietary fiber: an unmatched food component for sustainable health. Food Agric Immunol. (2024) 35:2384420. doi: 10.1080/09540105.2024.2384420

[113] Zhou Y Bai Z Wan K Qin T He R Xie C. Technology-based interventions on burden of older adults’ informal caregivers: a systematic review and meta-analysis of randomized controlled trials. BMC Geriatr. (2024) 24:398. doi: 10.1186/s12877-024-05018-w, 38704539 PMC11070124

[114] Ma X Liese AD Bell BA Martini L Hibbert J Draper C . Perceived and geographic food access and food security status among households with children. Public Health Nutr. (2016) 19:2781–8. doi: 10.1017/S1368980016000859, 27133939 PMC5588026

[115] Ganasegeran K Abdul Manaf MR Safian N Waller LA Abdul Maulud KN Mustapha FI. GIS-based assessments of neighborhood food environments and chronic conditions: An overview of methodologies. Annu Rev Public Health. (2024) 45:109–32. doi: 10.1146/annurev-publhealth-101322-031206, 38061019

[116] Tabrizi JS Azami-aghdash S Gharaee H. Public-private partnership policy in primary health care: a scoping review. J Prim Care Community Health. (2020) 11:11. doi: 10.1177/2150132720943769, 32842863 PMC7453464

[117] Gruzauskas V Burinskiene A Airapetian A Urbonaitė N. A geospatial framework of food demand mapping. Appl Sci. (2024) 14:6677. doi: 10.3390/app14156677

[118] Monaco A Verde L Filograna Pignatelli M Docimo A Ferrandes S Barrea L . Adherence to Mediterranean diet and prevalence of differentiated thyroid cancer: a single-center unit of thyroid surgery experience in a southern-Italy cohort. Minerva Endocrinol. (2025) 49. doi: 10.23736/S2724-6507.24.04173-3 39345029

[119] Lyerly R Rummo P Amin S Evans W Cohen ED Lawson E . Effectiveness of mobile produce markets in increasing access and affordability of fruits and vegetables among low-income seniors. Public Health Nutr. (2020) 23:3226–35. doi: 10.1017/S1368980020002931, 32886057 PMC10200560

[120] Hammon A Goralnik L. Barriers and bridges: contributions of mobile farmers markets to fresh food access for low-income and minority consumers. Local Environ. (2025) 30:447–62. doi: 10.1080/13549839.2024.2436024

[121] Kasprzak C Schoonover J Gallicchio D Haynes-Maslow L Vermont L Ammerman A . Using common practices to establish a framework for mobile produce markets in the United States. J Agric Food Syst Community Dev. (2021) 10:73–84. doi: 10.5304/jafscd.2021.104.029, 35548369 PMC9090202

[122] Atlanta Regional Commission. ARC Mini Grants Have an Outsized Impact on Food Insecurity Among Older Adults. Available online at: https://atlantaregional.org/news/aging-health/arc-mini-grants-have-an-outsized-impact-on-food-insecurity-among-older-adults/ (Accessed February 25, 2026).

[123] Thomas KS Bunker J Gadbois E Hilgeman M McCreedy E Mills W . Home-delivered meals and nursing home placement among people with self-reported dementia. JAMA Netw Open. (2023) 6:e2347195. doi: 10.1001/jamanetworkopen.2023.47195, 38117500 PMC10733798

[124] Guide to Community Preventive Services Nutrition: Home-delivered and Congregate Meal Services for Older Adults. (2021). Available online at: https://www.thecommunityguide.org/findings/nutrition-home-delivered-and-congregate-meal-services-older-adults (Accessed February 25, 2026).

[125] Zarei M Qorbani M Djalalinia S Sulaiman N Subashini T Appanah G . Food insecurity and dietary intake among elderly population: a systematic review. Int J Prev Med. (2021) 12:8. doi: 10.4103/ijpvm.IJPVM_61_19, 34084305 PMC8106269

[126] Sen K Laheji N Ramamonjiarivelo Z Renick C Osborne R Beauvais B. Examining the effect of contactless intergenerational befriending intervention on social isolation among older adults and students’ attitude toward companionship: content analysis. JMIR Aging. (2024) 7:e47908. doi: 10.2196/47908, 38175944 PMC10865196

[127] Fletcher J Jensen R. Mobile health: barriers to mobile phone use in the aging population. Online J Nurs Inform. (2015) 19

[128] Smyth SJ Webb SR Phillips PWB. The role of public-private partnerships in improving global food security. Glob Food Sec. (2021) 31:100588. doi: 10.1016/j.gfs.2021.100588, 38826717

[129] Fanzo J Shawar YR Shyam T Das S Shiffman J. Challenges to establish effective public-private partnerships to address malnutrition in all its forms. Int J Health Policy Manag. (2021) 10:934–45. doi: 10.34172/ijhpm.2020.262, 33619927 PMC9309966

[130] OASH. Public-Private Partnerships. Available online at: https://odphp.health.gov/foodismedicine/about-initiative/public-private-partnerships (Accessed 10 September 2024).

[131] Instacart. Instacart and Wellness West Announce Partnership to Tackle Food Insecurity on Chicago’s West Side. Available online at: https://investors.instacart.com/news-releases/news-release-details/instacart-and-wellness-west-announce-partnership-tackle-food (Accessed 24 October 2023).

[132] McCart A McCart D. Addressing food insecurity through municipal and community responses: a case study in Louisville, Kentucky. J Pub Health Issue Pract. (2025) 9. doi: 10.33790/jphip1100234

[133] OFMN. Ohio Senior Farmers Market Nutrition Program (SFMNP). Available online at: https://ohiofarmersmarketnetwork.org/senior-farmers-market-nutrition-program/ (Accessed 5 June 2025).

[134] National Council On Aging. Help to Gain Access to Healthy Food: Resources for Older Adults. Available online at: https://www.ncoa.org/article/help-to-gain-access-to-healthy-food-resources-for-older-adults/ (Accessed 19 May 2025).

[135] Kleinman A Chen H Levkoff SE Forsyth A Bloom DE Yip W . Social technology: an interdisciplinary approach to improving care for older adults. Front Public Health. (2021) 9. doi: 10.3389/fpubh.2021.729149, 35004562 PMC8733256

[136] Mennella C Maniscalco U De Pietro G Esposito M. Ethical and regulatory challenges of AI technologies in healthcare: a narrative review. Heliyon. (2024) 10:e26297. doi: 10.1016/j.heliyon.2024.e26297, 38384518 PMC10879008

[137] Fischer SH David D Crotty BH Dierks M Safran C. Acceptance and use of health information technology by community-dwelling elders. Int J Med Inform. (2014) 83:624–35. doi: 10.1016/j.ijmedinf.2014.06.005, 24996581 PMC4144164

[138] International Telecommunication Union. Measuring digital Development: Facts and Figures 2024. Geneva: ITU (2024).

[139] World Health Organization. Global Strategy on digital Health 2020–2025. Geneva: World Health Organization (2021).

[140] Yang R Gao S Jiang Y. Digital divide as a determinant of health in the U.S. older adults: prevalence, trends, and risk factors. BMC Geriatr. (2024) 24:1027. doi: 10.1186/s12877-024-05612-y, 39709341 PMC11662839

[141] Levine DM Lipsitz SR Linder JA. Trends in seniors’ use of digital health technology in the United States, 2011-2014. JAMA. (2011) 316:538. doi: 10.1001/jama.2016.9124, 27483069

[142] Ernsting C Dombrowski SU Oedekoven M O’Sullivan JL Kanzler E Kuhlmey A . Using smartphones and health apps to change and manage health behaviors: a population-based survey. J Med Internet Res. (2017) 19:e101. doi: 10.2196/jmir.6838, 28381394 PMC5399221

[143] Campos-Castillo C Anthony D. Racial and ethnic differences in self-reported telehealth use during the COVID-19 pandemic: a secondary analysis of a US survey of internet users from late march. J Am Med Inform Assoc. (2021) 28:119–25. doi: 10.1093/jamia/ocaa221, 32894772 PMC7499625

[144] Lee SR Maxi A Kim L Kim Y Choe I Hong C . Enhancing telehealth accessibility for older adults in underserved areas: a 4M framework approach. Gerontol Geriatr Med. (2024) 10:10. doi: 10.1177/23337214241277045, 39286401 PMC11403561

[145] Xie B. Older adults, computers, and the internet: future directions. Geron. (2003) 2:289–305. doi: 10.4017/gt.2003.02.04.002.00

[146] Fozard JL Wahl H-W. Age and cohort effects in gerontechnology: a reconsideration. Geron. (2012) 11. doi: 10.4017/gt.2012.11.01.003.00

[147] Fitzpatrick PJ. Improving health literacy using the power of digital communications to achieve better health outcomes for patients and practitioners. Front Digit Health. (2023) 5. doi: 10.3389/fdgth.2023.1264780, 38046643 PMC10693297

[148] Grosman-Rimon L Wegier P. With advancement in health technology comes great responsibility – ethical and safety considerations for using digital health technology: a narrative review. Medicine. (2024) 103:e39136. doi: 10.1097/MD.0000000000039136, 39151529 PMC11332755

[149] Haimi M. The tragic paradoxical effect of telemedicine on healthcare disparities- a time for redemption: a narrative review. BMC Med Inform Decis Mak. (2023) 23:95. doi: 10.1186/s12911-023-02194-4, 37193960 PMC10186294

[150] Odoms-Young A Brown AGM Agurs-Collins T Glanz K. Food insecurity, neighborhood food environment, and health disparities: state of the science, research gaps and opportunities. Am J Clin Nutr. (2024) 119:850–61. doi: 10.1016/j.ajcnut.2023.12.019, 38160801 PMC10972712

[151] Schroeder T Dodds L Georgiou A Gewald H Siette J. Older adults and new technology: mapping review of the factors associated with older adults’ intention to adopt digital technologies. JMIR Aging. (2023) 6:e44564. doi: 10.2196/44564, 37191976 PMC10230357

[152] Rodrigues AM Gregório MJ Gein P Eusébio M Santos MJ de Sousa RD . Home-based intervention program to reduce food insecurity in elderly populations using a TV app: study protocol of the randomized controlled trial Saúde.Come senior. JMIR Res Protoc. (2017) 6:e40. doi: 10.2196/resprot.6626, 28288956 PMC5368350

[153] Chen K Chan A. Use or non-use of Gerontechnology—a qualitative study. Int J Environ Res Public Health. (2013) 10:4645–66. doi: 10.3390/ijerph10104645, 24084674 PMC3823313

[154] Kaboré SS Ngangue P Soubeiga D Barro A Pilabré AH Bationo N . Barriers and facilitators for the sustainability of digital health interventions in low and middle-income countries: a systematic review. Front Digit Health. (2022) 4. doi: 10.3389/fdgth.2022.1014375, 36518563 PMC9742266

[155] Lin J Bates SM Allen LN Wright M Mao L Kidd M. Integrating Mobile health app data into electronic medical or health record systems and its impact on health care delivery and patient health outcomes: scoping review. JMIR Mhealth Uhealth. (2025) 13:e66650–15. doi: 10.2196/66650, 40549960 PMC12208509

[156] Lo FP-W Qiu J Jobarteh ML Sun Y Wang Z Jiang S . AI-enabled wearable cameras for assisting dietary assessment in African populations. NPJ Digit Med. (2024) 7:356. doi: 10.1038/s41746-024-01346-8, 39638852 PMC11621677

[157] Gao L Wang C Wu G. Wearable biosensor smart glasses based on augmented reality and eye tracking. Sensors. (2024) 24:6740. doi: 10.3390/s24206740, 39460220 PMC11511461

[158] Stankoski S Kiprijanovska I Gjoreski M Panchevski F Sazdov B Sofronievski B . Controlled and real-life investigation of optical tracking sensors in smart glasses for monitoring eating behavior using deep learning: cross-sectional study. JMIR Mhealth Uhealth. (2024) 12:e59469. doi: 10.2196/59469, 39325528 PMC11467608

[159] Swetha P Balijapalli U Feng S-P. Wireless accessing of salivary biomarkers based wearable electrochemical sensors: a mini-review. Electrochem Commun. (2022) 140:107314. doi: 10.1016/j.elecom.2022.107314

[160] Malon RSP Sadir S Balakrishnan M Córcoles EP. Saliva-based biosensors: noninvasive monitoring tool for clinical diagnostics. Biomed Res Int. (2014) 2014:1–20. doi: 10.1155/2014/962903, 25276835 PMC4172994

[161] Cohen R Fernie G Roshan Fekr A. Fluid intake monitoring systems for the elderly: a review of the literature. Nutrients. (2021) 13:2092. doi: 10.3390/nu13062092, 34205234 PMC8233832

[162] Kalantarian H Alshurafa N Le T Sarrafzadeh M. Monitoring eating habits using a piezoelectric sensor-based necklace. Comput Biol Med. (2015) 58:46–55. doi: 10.1016/j.compbiomed.2015.01.005, 25616023

[163] Abeltino A Riente A Bianchetti G Serantoni C De Spirito M Capezzone S . Digital applications for diet monitoring, planning, and precision nutrition for citizens and professionals: a state of the art. Nutr Rev. (2025) 83:e574–601. doi: 10.1093/nutrit/nuae035, 38722240 PMC11986332

[164] Gioia S Vlasac IM Babazadeh D Fryou NL Do E Love J . Mobile apps for dietary and food timing assessment: evaluation for use in clinical research. JMIR Form Res. (2023) 7:e35858. doi: 10.2196/35858, 37327038 PMC10337248

[165] Aure CF Kluge A Moen A. Older adults’ engagement in technology-mediated self-monitoring of diet: a mixed-method study. J Nurs Scholarsh. (2021) 53:25–34. doi: 10.1111/jnu.12619, 33316147 PMC7839486

